# An Anthropological Perspective: Another Dimension to Modern Dental Wear Concepts

**DOI:** 10.1155/2012/741405

**Published:** 2012-12-10

**Authors:** John A. Kaidonis, Sarbin Ranjitkar, Dimitra Lekkas, Grant C. Townsend

**Affiliations:** School of Dentistry, The University of Adelaide, Adelaide, SA 5005, Australia

## Abstract

For many years, research on tooth wear by dental academics has been diametrically opposite to that of anthropological research, with each discipline having a different understanding as to the nature of the wear processes. Dental focus revolved around preventive and restorative considerations while the anthropological focus was a biological understanding related to human evolution, diet, environment, form, and function and included all the craniofacial structures. Introducing the anthropological perspective into modern dentistry gives an insight into the “bigger picture” of the nature and extent of tooth wear. By combining anthropological evidence with clinical knowledge and experience, it is most likely to provide the best-informed and biologically based approach to the management of tooth wear in modern societies.

## 1. Introduction

The current mechanisms of tooth wear that are generally accepted within the dental literature include abrasion, erosion, and attrition. Abrasion occurs from the friction of any foreign substance forced over tooth surfaces (e.g., toothbrush, food); erosion is a modern-day condition resulting from the dissolution of tooth substance by acids that do not originate from oral biofilms, and attrition results from tooth grinding without the presence of food. Although it is not the purpose of this paper to detail the specifics of each mechanism, it can be said that abrasion caused by food generally acts more broadly on a tooth surface eventually causing dentinal scooping, whereas the characteristic feature of attrition is the production of well-defined facets on opposing teeth. Erosion can act on nonoccluding surfaces, does cause dentinal scooping, and can be distinguished clinically by the characteristic appearance of a glazed affected surface [1]. The microwear detail resulting from these mechanisms enables them to be distinguished from one another reasonably easily, particularly if one of the mechanisms is dominant. However, they often act together with different intensities and durations, making it difficult to differentiate between them [2].

In addition to the mechanisms described above, abfraction can also be included as a mechanism of tooth loss. Although currently controversial, it nevertheless may be considered as an added factor contributing to noncarious cervical lesions [3–6]. Erosion and abfraction are certainly modern-day conditions, while abrasion and attrition have been described in different species and phyla throughout the course of vertebrate evolution. 

Human tooth wear in precontemporary populations has been studied by physical anthropologists before the last century. Efforts to find associations between tooth wear and culture, gender, age, craniofacial geometry, tooth size, dental crown traits, diet and environment, and so forth, have provided many new insights over the years [7–9]. In contrast, there is relatively little information about the extent and nature of genetic and environmental contributions to variation in tooth grinding [10]. 

Anthropological understanding of tooth wear in precontemporary populations was focused, mainly, for many years, on the abrasiveness of food, cultural practices, and the use of teeth as tools [7]. The mechanisms of attrition and erosion, according to today's definitions, were not considered in any detail. Unfortunately, the terms were often used synonymously by anthropologists, referring to wear caused by an abrasive diet, thereby adding some confusion in the literature.

Furthermore, anthropologists considered tooth wear as a normal physiological process, with the teeth in most individuals remaining functional throughout life. The changes to tooth surfaces and to the dental occlusion resulted in a series of cascading adaptive processes involving the whole stomatognathic system [11]. It was only when the teeth became so worn that they were nonfunctional, that a pathological state was considered to be present. 

In contrast to the anthropologist's perspective, tooth wear research from a dentist's viewpoint has traditionally focused on restorative management and it has only become a major topic of interest among dentists during the last 20 years or so. Much of the research in this area nowadays concentrates on improving understanding and management of modern-day conditions (e.g., erosion). Some dental researchers, especially those who trained according to “gnathological” principles, have maintained a more restricted mechanical view of tooth wear and considered any evidence of wear to be pathological [12]. This dichotomy between anthropological and dental research reflects the origins and nature of each discipline; however, both disciplines should be viewed as components of a “bigger picture.” 

Contemporary populations show little abrasive wear because of the consumption of processed, softer food. Therefore, much of the wear in our modern societies results from erosion and attrition. While attrition is common in current populations, there is evidence that attrition caused by tooth grinding may have always been a common behaviour in humans [13].

In summary, without an awareness and understanding of the considerable amount of anthropological research that has been carried out relating to tooth wear, many dentists retain a general impression that today's mostly unworn dentitions represent the “norm,” when in fact the post-industrial human dentition and occlusion tends to represent a neotenous stage of development. That is, it tends to present in a similar manner to the relatively unworn dentition of a young Paleolithic individual. In addition to the anthropological approach, paleontological studies associating dental wear with dental and craniofacial anatomy, the evolution of cusps, form and function, and animal behavior also add insights to the “bigger picture” of the nature and extent of tooth wear.

## 2. Precontemporary Populations

There are many “interesting” dental features that have been described and documented by anthropologists that express the true dynamic nature of the craniofacial skeleton and dental occlusion. Some of these features will be briefly described to indicate the nature and extent of variation that can be observed in the stomatognathic system from both spatial and temporal perspectives. Many dental practitioners are unaware of these features and they are generally not considered in any depth, if at all, in dental curricula.

### 2.1. Interproximal Wear

Interproximal wear occurs when the proximal surfaces of teeth rub together. The differential loads acting on teeth cause them to move independently from one another during function. The resulting interproximal wear is proportional to the observed occlusal wear and therefore the load subjected on the teeth [14]. When teeth erupt, early interproximal contact points eventually become contact areas and the contacts between adjacent teeth remain closed due to mesial drift. This results in a reduction in dental arch length, the amount of which is proportional to the degree of wear [15–17]. This observation also underpinned the theory of attritional occlusion [16], which proposed that interproximal wear reduced the prevalence of dental crowding and third molar impactions as more room was created within the dental arch. This theory, when extrapolated to modern orthodontics, was also partly used as a rationale for the extraction of premolar teeth during orthodontic management of crowded dentitions.

**Figure 1:**
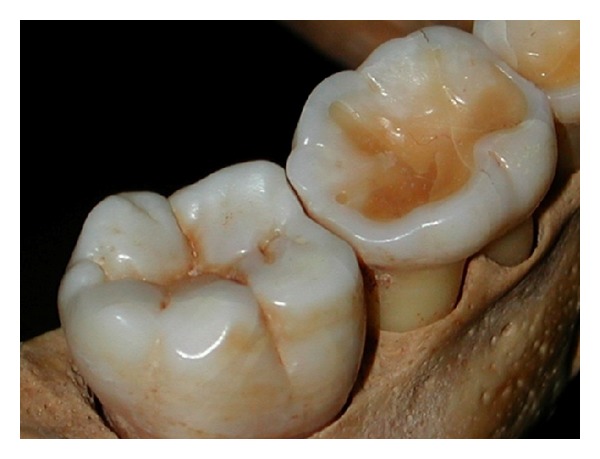
Interproximal wear in a young child. The mesial of the permanent first lower molar wears faster than the distal of the primary second molar.

Commonly, with interproximal wear, the mesial surfaces of teeth wear faster than the distal surfaces of adjacent teeth resulting in the development of mesial concavities (Figure 1). These tend to be more evident in dentitions exhibiting advanced wear [14]. Explanations for this characteristic wear pattern have been proposed to involve the distribution of force vectors through the teeth under load [18]. Although this type of wear is often considered to be insignificant by dental practitioners, there is strong evidence that dental occlusions do change over time through a dynamic process that anthropologists consider to be quite physiological.

### 2.2. Interproximal Grooving

**Figure 2:**
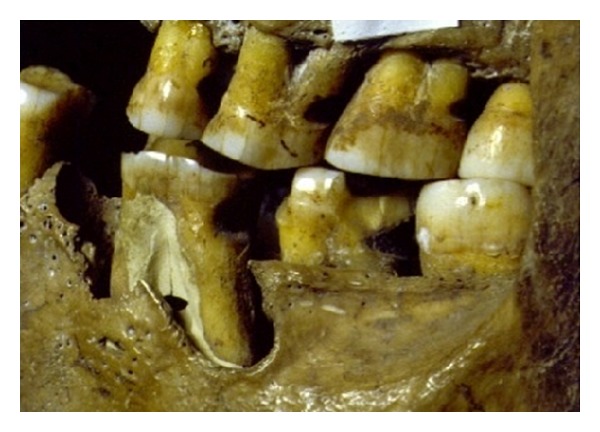
The passing of kangaroo tendon between posterior teeth caused distal abrasive wear called “interproximal grooving.”

Interproximal grooving can be considered to be a type of noncarious interproximal lesion most often seen on the distal surfaces of posterior teeth in hunter-gatherer populations, particularly Australian Aborigines (Figure 2). This type of abrasive wear results from cultural practices where the teeth are used as tools. For example, at times when kangaroo tendon was chewed and passed between the teeth, it could slip interproximally and wear the distal surface of a tooth as the tendon was pulled anteriorly [19].

### 2.3. X-Occlusion

**Figure 3:**
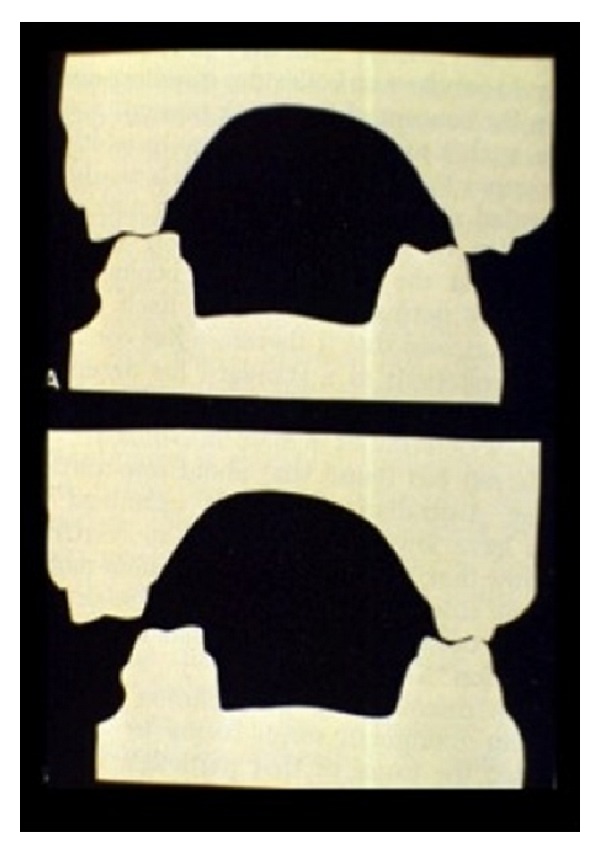
An example of “X-occlusion” or “alternate intercuspation.” The plaster models sectioned coronally across the molars highlight the two types of centric occlusions.

“X-occlusion” or “alternate intercuspation” are two terms used to define a characteristic mode of occlusion observed among Australian Aborigines living in their traditional lifestyle (Figure 3). Here, the dental arches exhibit two different centric occlusions resulting from the upper arch being wider than the lower. When there is maximum intercuspation on the left side, the right side displays a large overjet, and vice versa [20]. The dentitions of these individuals are fully functional yet, according to “modern” dental opinion, such dentitions would generally be considered nonfunctional and orthodontic treatment would likely be recommended. This is a good example of how opinion about dental issues is often dependent on the breadth and depth of one's educational background.

### 2.4. Helicoidal Plane

**Figure 4:**
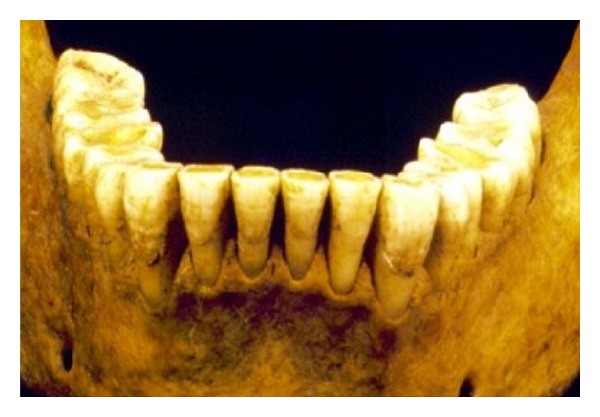
The helicoidal plane. The wear pattern produces an occlusal twist of the molar teeth. The buccal inclination on the first molars changes to a neutral inclination on the second molars that in turn changes to a lingual inclination on the third molars.

This occlusal characteristic has been documented by anthropologists in different human populations and has been described as a posterior occlusal twist of worn occlusal surfaces [21, 22]. Here, the occlusal surfaces of the lower first molars slope towards the buccal, the second molars are horizontal, and the third molars slope lingually (Figure 4). The opposing teeth follow the reverse pattern. This alteration in the occlusal curvatures can also occur in modern societies where there is advanced dental wear, but these changes often go unnoticed.

### 2.5. Form and Function, Differential Wear, and Occlusion

Contrary to belief within some dental circles, dental occlusions are dynamic and continually changing. Tooth wear, particularly abrasion in precontemporary hunter-gatherer populations, is the main mechanism that changes the morphology of teeth over time. A “canine-protected” occlusion during youth tends to change progressively into “group function,” causing a number of consequential adaptive changes [23]. As the wear progresses, continual eruption of teeth compensates for the wear (a feature seen commonly in other nonhuman species as well). The established occlusal vertical dimension at any given point of time is, therefore, a by-product of these two opposing processes. As the cusps reduce in height and disappear, the “tear-drop” cycle of the masticatory stroke becomes wider and, with excessive wear, corresponding remodeling of the glenoid fossa also tends to occur [24]. 

Exposure and scooping of the dentine by abrasion (leading to differential wear) is important in maintaining physiological function, often described as a scissorial action, supported by a wider “tear-drop” shape of the horizontal aspect of the masticatory stroke. Scissorial point cutting is a common evolutionary feature across different species and phyla. This feature has been described in the literature, is universal among herbivores, and is also strongly evident in human and nonhuman primates [2, 25].

The scooping of dentine allows the resulting, less-worn enamel to act as a sickle-shaped blade that moves against a similar blade facing in the opposite direction in the opposing arch. An extension to this well-documented account, which explains how teeth not only grind but actually cut food, is the theory of thegosis. This theory was developed over 50 years ago by Ron Every, whose observations of different species (including humans) identified that tooth grinding was a sharpening mechanism of the sickle blades resulting from differential wear to enhance the masticatory efficiency [26]. In addition, Every extended his observations to specialisations within other species (e.g., baboons, wild boars), and showed how tooth grinding occurred during stressful encounters (fight-or-flight response) often in order to sharpen the canines—their biological weapons. This theory was further extended to the notion that tooth grinding in humans is built into our genetic codes, and is, therefore, an instinctive universal behavior [25, 27–30]. 

Studies involving differential wear seem to indicate that dentine exposed by abrasive action is not usually sensitive because a mechanical smear layer forms on the tooth surface, thereby occluding dentinal tubules. When dentine is found to be sensitive, there are open dentinal tubules caused by superimposed erosion. In addition, studies on width-versus-depth ratios of scooped dentine show that abrasive scooping tends to be shallower than that caused by erosion [31]. 

Tooth wear mechanisms, such as attrition and in particular abrasion, have been present since the reptilian-mammalian transition. These mechanisms were a selective force that was responsible for many adaptive evolutionary changes, as well as masticatory specialisations, specific for each species.

There is an acknowledged common pattern of wear observed between opposing posterior teeth in most species. The buccal cusps of the lower molars wear faster than the lingual cusps, while the palatal cusps of the upper molars wear faster than the buccal cusps. Dentists and anthropologists agree that a “tear-drop” masticatory action, together with a power-stroke, can explain this pattern of wear. Interestingly, accessory cusps are seen on the palatal surfaces of the upper teeth (e.g., Carabelli trait) and on the buccal cusp of the lower molars (protostylids) [32]. These accessory cusps are able to “take over” the load as the working cusps wear. This example shows how tooth wear, from an evolutionary perspective, is a selective force that is likely to have been responsible for changes in dental morphology. 

Differences in the extent and pattern of tooth wear have often been observed between the dentitions of precontemporary populations. These relate commonly to environmental differences in diet; however, social and cultural determinants can also contribute to these differences. For example, there are recorded differences in wear patterns between males and females in some precontemporary populations because of differences in the tasks undertaken by each sex [33]. Some societies even “file down” anterior teeth for aesthetic reasons or have teeth avulsed as a cultural practice [34].

## 3. Contemporary Populations

**Figure 5:**
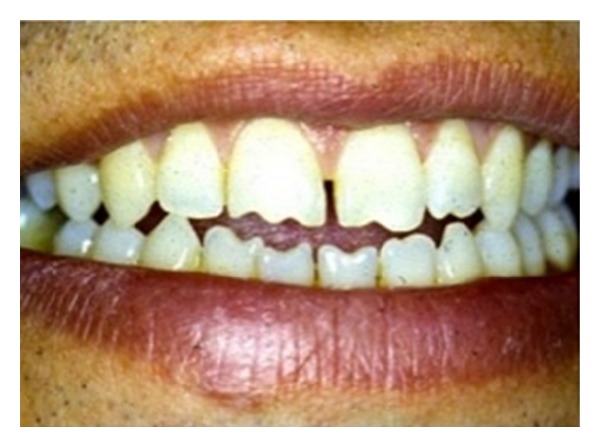
The “vertical” positioning and crushing of pumpkin and watermelon seeds using the anterior teeth has resulted in the abrasion “notches” in those teeth.

So-called contemporary, modern or post-industrial populations tend to show wear characteristics that are different to those of our ancestors. As a general rule, the wear caused by abrasive food is much reduced in modern societies because of our softer, more processed diets. However, there are still dietary differences between current populations where the preparation and consumption of specific foods can result in nontypical abrasion patterns.  Figure 5 displays a specific abrasive wear pattern caused by the crushing of dried pumpkin and watermelon seeds between the incisors.

### 3.1. Attrition

**Figure 6:**
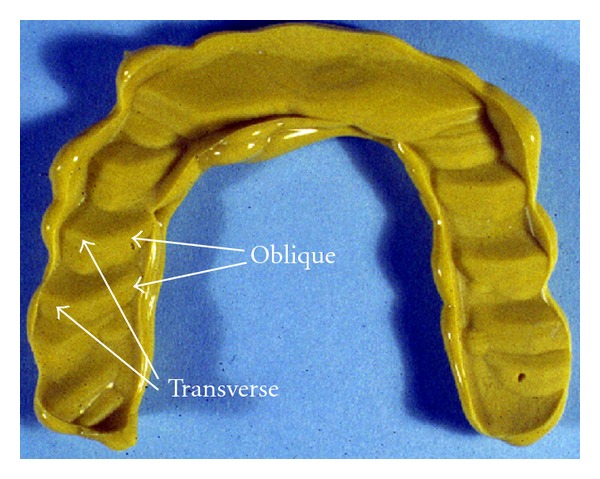
An impression of an occluding surface of an excessively worn night guard. The transverse and oblique wear patterns indicate the lateral mandibular movement that formed them. In clinical and micrographic assessments, the wear pattern would be evident as striations.

**Figure 7:**
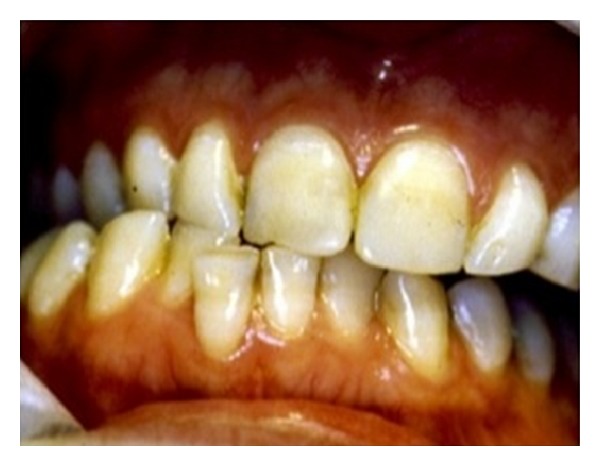
An extreme lateral mandibular movement past the canine edge-to-edge has resulted in the notch on the upper right central incisor.

Microwear patterns within facets characteristically have parallel striations that are orientated in a horizontal or oblique direction on posterior teeth and in an anterior-lateral direction on anterior teeth. This microwear patterning has been described in the past leading to two diametrically opposing conclusions. One conclusion states that the microwear patterns have resulted from mandibular action during mastication [35], while the other conclusion states that tooth grinding is the cause of these patterns [27]. Recent studies indicate that tooth grinding is indeed the most likely mechanism involved with these patterns, as we have observed the same patterns on the occlusal surfaces of night guards (Figure 6). This view is also supported by case studies that show wear patterns resulting from eccentric mandibular movements (Figure 7).

The mandibular movement during grinding results from a lateral movement where the lower canine tends to ride along the palatal surface of the upper canine and past the canine-edge-to-edge position into an eccentric position. Here, one condyle pivots at the postglenoid tubercle (ipsilateral), while the contralateral condyle moves anteriorly, medially and inferiorly along the condylar eminence due to the unilateral action of the lateral pterygoid muscle. The resulting microwear striations are generally seen on the wear facets of anterior teeth, but they also become evident on the posterior teeth where the group function is present. These striations have been described as transverse and oblique on posterior teeth and anterolateral on anterior teeth [27].

Often, unusual wear patterns can be observed in dental surgeries where anterior teeth have worn much faster than posterior teeth. Although further research is required to tease out the reasons for these differences, it appears that a patient's craniofacial morphology, together with their occlusal scheme, plays a role. For example, an Angle Class 2, Division 2 (or a deep anterior bite) malocclusion tends to be associated with excessive anterior tooth wear. The simple geometry of having a deep bite implies that a canine-protected occlusion will continue for a longer period of time, thereby exacerbating the wear on the anterior teeth.

### 3.2. Social, Cultural, Iatrogenic, and Other Determinants

As in past populations, modern societies are also influenced by prevailing fashions, often with severe consequences. Tongue and lip piercing fall into this category, commonly causing mechanical damage to adjacent teeth. 

Similarly, the dental bur has been responsible for iatrogenic dental wear when occlusal equilibrations have been performed in dental practices. Although there are differences of opinion about the need for occlusal equilibrations and the resulting wear has been considered by some operators to be relatively minor, these procedures, nevertheless, remove tooth structure irreversibly. 

**Figure 8:**
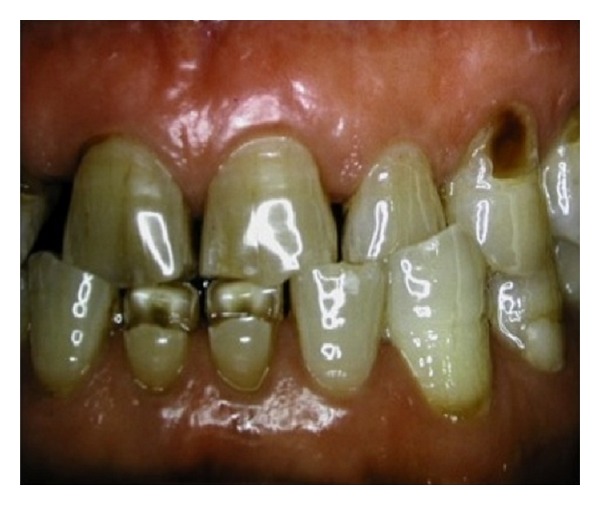
Two wedge-shaped lesions have formed on the lower first central incisors. To date there is no definite explanation as to the cause of the wear pattern.

There are occasions when tooth wear patterns are observed without any obvious explanation, even after thorough questioning of the patient. An example is depicted in Figure 8 where wedge-shaped lesions were observed on the worn incisal edges of lower central incisors.

## 4. Conclusion

Tooth wear patterns vary within and between human populations, both past and present, for a variety of reasons. The understanding and opinions formed about the nature and extent of tooth wear within the human dentition still tend to reflect the training and educational background of the observer. In our view, combining anthropological evidence with clinical knowledge and experience is most likely to provide the best-informed and biologically based approach to the management of tooth wear in modern societies. The anthropological evidence introduces a broader view of the pattern and extent of tooth wear within and between populations, allowing for a “rethink” of some of our current dental concepts.
